# Postoperative pain after single visit root canal treatment using domestic vs. medical grade sodium hypochlorite: a randomized controlled trial

**DOI:** 10.1186/s12903-026-08185-x

**Published:** 2026-04-13

**Authors:** Maha Nasr, Nermine Hassan

**Affiliations:** 1 Lecturer in Endodontics, Faculty of Oral and Dental Medicine, Egyptian Russian University, Badr City, Egypt; 2 Lecturer in Endodontics, Faculty of Dentistry, Cairo University, Cairo, Egypt

**Keywords:** Sodium Hypochlorite, Postoperative Pain, Symptomatic irreversible pulpitis, Irrigation, Single visit, Root canal treatment

## Abstract

**Background:**

Sodium hypochlorite (NaOCl), the primary endodontic disinfectant, is available in both domestic-grade products and specialized medical-grade formulations. The stability and concentration of domestic-grade products are inconsistent, potentially affecting safety and clinical outcomes. This trial compared the impact of domestic-grade versus medical-grade NaOCl on postoperative pain following endodontic treatment in patients with symptomatic irreversible pulpitis.

**Methodology:**

Sixty participants with symptomatic irreversible pulpitis in mandibular molars were randomly distributed into two groups (*n* = 30). Following instrumentation with Edge X7 rotary files, Group A received domestic-grade NaOCl irrigation, while Group B was treated with a medical-grade formulation. Participants recorded pain intensity on an 11-point numerical rating scale (NRS) at 6, 12, 24, 48, and 72 h.

**Results:**

Domestic-grade NaOCl was associated with significantly higher pain levels at 6 h (median 3 vs. 0; *p* < 0.05) and 12 h (median 2 vs. 0; *p* < 0.05) compared to medical-grade NaOCl. No significant differences were observed at 24, 48, or 72 h.

**Conclusions:**

Domestic bleach caused significantly higher early postoperative pain than medical-grade sodium hypochlorite due to its unstandardized, higher concentration. Standardized medical-grade irrigants are recommended to maximize patient comfort and clinical predictability.

**Trial Registration:**

The protocol for this prospective, parallel group, double blinded, randomized clinical trial was registered at ClinicalTrials.gov (https://register.clinicaltrials.gov/) on 18/11/2025, registration number (NCT07248189). The trial was retrospectively registered.

**Supplementary Information:**

The online version contains supplementary material available at 10.1186/s12903-026-08185-x.

## Background

Postoperative pain after endodontic procedures is an undesirable yet common outcome influenced by factors such as preoperative pain, microbial, chemical, and mechanical injury to the pulp and or periradicular tissues [1]. The most common cause is acute periradicular inflammation, with severity proportional to the extent of tissue injury. Mild discomfort is common even when procedures meet high standards, whereas flare-ups involving severe pain and swelling are rare, reported in 1.4%–16% of cases [2].

For both patients and operators, preventing post-endodontic discomfort is crucial. Postoperative pain is frequently experienced after root canal therapy, especially in teeth with nonvital pulps. The extrusion of infected fluids and debris is a frequent cause [3]. Reports indicate postoperative endodontic pain occurs in 24–40% of cases on average and up to 80% within the first 24 h [4].

For decades NaOCl, as the primary choice for root canal irrigation, has played a central role in treating primary and secondary endodontic infections. Besides having a broad-spectrum antimicrobial activity, it is the only irrigant proven to have tissue-dissolving ability. This makes its use necessary in all cases for disinfection [5].

NaOCl, a widely used disinfectant, is available in domestic-grade formulations and special medical-grade dedicated dental formulations. Both formulations are used safely for irrigation and endodontic disinfection [6, 7]. However, the production of NaOCl involves an electrochemical process that can introduce contaminants, such as iron and other heavy metals, especially from the electrodes used. Household bleach, a commonly available form of NaOCl, may contain undisclosed levels of these metals and various additives, including polyacrylates intended to bind contaminants. In contrast, pharmaceutical-grade NaOCl solutions are manufactured under stringent controls, utilizing purified water and filtration procedures to minimize impurities and regulate metal content [8].

Additionally, the stability and concentration of domestic-grade NaOCl products are questionable and can affect the disinfecting ability and safety of those formulations for dental use [7]. While there is widespread in vitro evidence regarding the variable free chlorine concentration, pH, and chemical purity of commercial bleaches, there is a lack of clinical evidence determining the impact of these variables on patient-centered outcomes, such as postoperative pain. This study aims to fill that gap by comparing the clinical performance of a standardized medical-grade formulation against a domestic-grade product in a controlled clinical setting.

## Materials and methods

### Study design, setting and recruitment

**Fig. 1:**
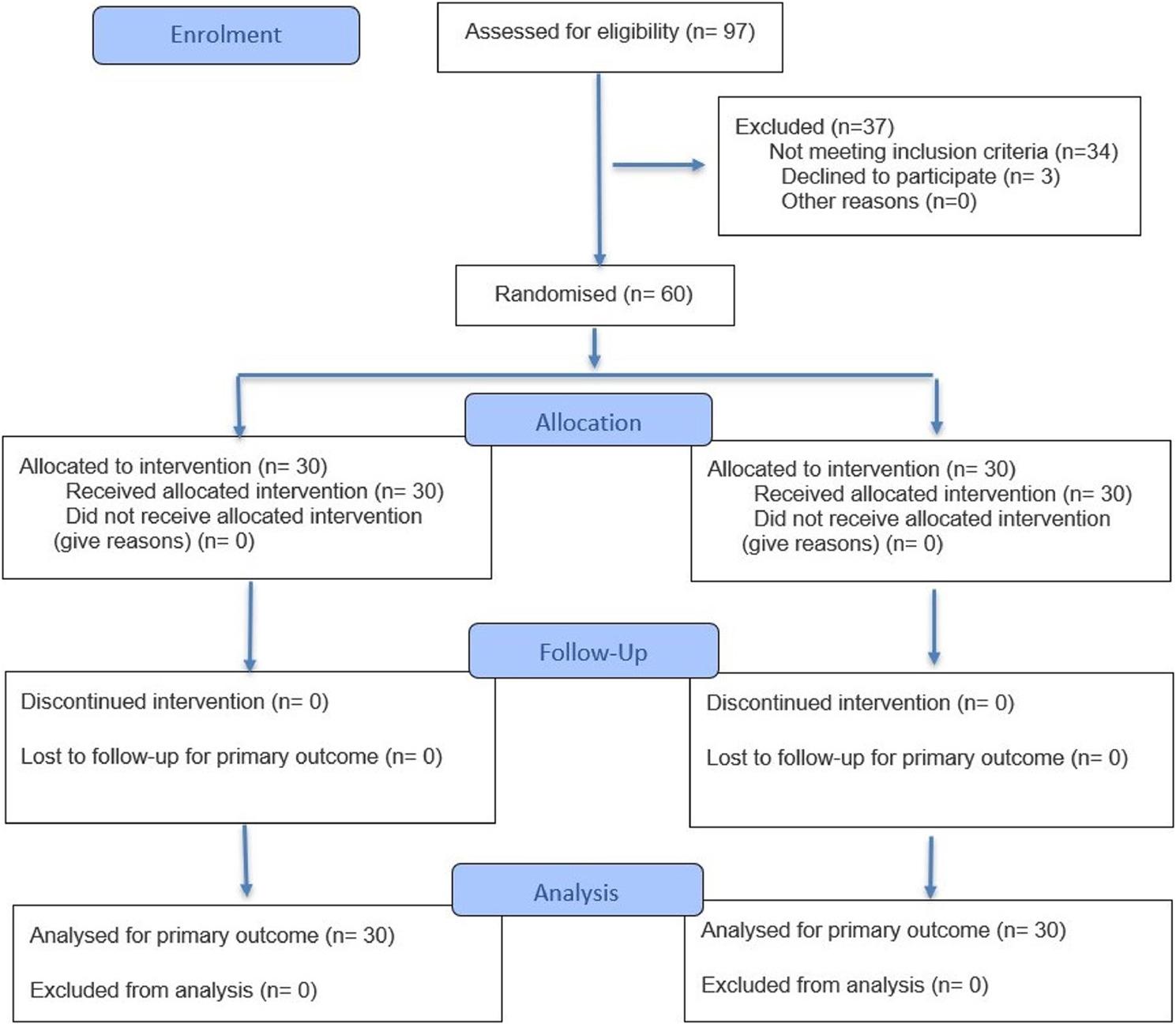
(CONSORT 2025 Flow Diagram). Flow diagram of the progress through the phases of the trial for the two groups

This was a prospective, two-arm, parallel-group, double-blinded, randomized controlled clinical trial. The study received ethical approval from the Research Ethical committee at the Faculty of Oral and Dental Medicine, Egyptian Russian University number [D-ERU-REC (33)], and was registered retrospectively at ClinicalTrials.gov (https://register.clinicaltrials.gov/) number (NCT07248189). Potential subjects were provided with a comprehensive explanation regarding the study’s purpose, clinical procedures, and potential risks. Written consent forms were signed by all individuals who agreed to participate in the trial. The study was conducted in accordance with the guidelines of the World Medical Association Declaration of Helsinki, and the Institutional ethical committee. Participants were recruited from the outpatient clinic, Department of Endodontics, Oral and Dental Medicine, Egyptian Russian University between October and December, 2025. The trial was reported in accordance with the Consolidated Standards of Reporting Trials (CONSORT) [9] and the progress through the phases of the trial of is represented in Fig. 1.

### Sample size calculation

Sample size calculation was performed using G*Power software (version 3.1.9.7). Based on a pilot study of 10 participants per group, the primary outcome variable—postoperative pain intensity on the 11-point NRS—showed a large effect size (d = 1.2311583). This high effect size reflected a substantial clinical difference in early postoperative pain (6 and 12 h) between the medical and domestic-grade groups during the pilot phase. To detect a significant difference with 98% power and a 5% significance level, 24 participants per group were required. The total was increased to 60 (30 per group) to account for a potential 25% dropout rate.

Given the clinical relevance of post-operative pain outcomes following endodontic treatment, a higher power (98%) was chosen to reduce the likelihood of false negative results, 90% power gives total sample size of 26 + 25% drop out (Total 34, 17 per group).

### Randomization, allocation concealment, and blinding

Trial participants were assigned to one of the two study arms in a 1:1 ratio based on a sequence generated via a computer-based random number program (random.org) as follows: Group A: Domestic-grade NaOCl and Group B: Medical-grade NaOCl, the randomization sequence was generated by an independent investigator who was not involved in patient recruitment, clinical procedures, or outcome assessment. The sodium hypochlorite solutions utilized in this study included a specialized medical-grade irrigant (JK Dental Vision, Egypt; Lot No. 037, MFG 8/2025) and a domestic-grade variant (Clorox, Egypt; Batch No. B-40, MFG 17/07/2025). To ensure allocation concealment, assignments were concealed within sequentially numbered, opaque, sealed envelopes. These envelopes contained the group designation written on folded slips of paper and were opened only by each patient at the time of treatment. Both the participants and the operator were blinded to the interventions. As outlined in the study’s ethical consent, the treatment details were shared with participants without revealing the irrigant type.

Postoperative pain outcomes were self-reported by patients using standardized pain diaries. Data entry and statistical analysis were conducted by an independent investigator blinded to group allocation. Blinding was maintained until completion of data analysis.

### Preparation of the irrigating solutions

A half - concentration (≈ 2.5%) solution was freshly prepared by the non-treating operator just before the participants were allocated to either group A or B, by adding equal amounts of each solution and distilled water into the container to be used for irrigation throughout the mechanical preparation. To maintain blinding, the non-treating operator transferred both irrigating solutions were into identical, neutral, coded containers prior to use, while the treating clinician remained unaware of the irrigant type throughout the procedure.

### Eligibility criteria

Healthy adult patients between ages 18 and 48 years diagnosed with symptomatic irreversible pulpitis in one mandibular molar were considered eligible and approached by the dental emergency clinic attending clinician. There were no gender restrictions. Exclusion criteria included patients with asymptomatic irreversible pulpitis, having periapical radiolucency, pregnant or lactating females, patients who had been taking pain medication 12 h earlier and, those with contributory medical history (ASA > II). Study eligibility was also limited to individuals with only one symptomatic tooth in a given mandibular quadrant, thus, ensuring that pain assessments were localized and accurate.

### Treatment procedures

Personal, medical, and dental histories, including the chief complaint, were collected using standardized forms. Extra-oral and intra-oral examinations were then performed, and the offending tooth was assessed clinically with a mirror and probe. Periapical radiographs were taken to detect periodontal ligament widening, periapical radiolucency, and deep or recurrent caries. Clinical confirmation of symptomatic irreversible pulpitis relied on a comprehensive review of patient history paired with diagnostic testing. Specifically, an intensified reaction to thermal (cold) stimuli, when compared to the healthy contralateral control, and positive electric pulp test results in cases where radiographs indicated minimal or no periodontal ligament (PDL) space widening. All root canal treatment procedures were performed in a single visit by an experienced endodontist with > 10 years of clinical and academic experience. Each patient was provided with a numerical rating scale (NRS-11) chart to record their preoperative pain level. The tooth was anesthetized with 1.8 mL of 4% articaine with 1:100,000 epinephrine (Artinibsa; Inibsa, Spain). Access cavity preparation was performed using a size 4 round bur and Endo-Z bur (Dentsply Maillefer, Switzerland). The tooth was then isolated using a rubber dam, and the working length was determined using an apex locator (Root ZX, J. Morita, USA) and radiographically confirmed to be 0.5–1 mm short of the radiographic apex. Patients were randomly assigned to two groups: Group A, in which domestic-grade NaOCl was used, and Group B, in which medical-grade NaOCl was used. Chemomechanical preparation was performed with the Edge X7 nickel-titanium (NiTi) rotary system (Edge-Endo, USA) in strict adherence to the manufacturer’s protocol. Root canals were irrigated with 3 mL of the assigned solution after each instrument change, delivered using a 29-gauge side vented needle (NaviTip; Ultradent, South Jordan, UT) positioned 1 mm short of the working length at a flow rate of 3 mL per minute to avoid any risk of irrigant extrusion. A total volume of approximately 15 mL of either domestic-grade NaOCl or medical-grade NaOCl was used per canal. Following instrumentation, 5 mL 17% ethylenediaminetetraacetic acid (EDTA) (Endo-solution, Cerkamed, Poland), was used for 1 min for smear layer removal, followed by 5 mL distilled water, 5 mL NaOCl as per group assignment and a final flush with 10 mL distilled water. All canals were dried and filled using 0.04 taper gutta-percha and resin sealer (ADSEAL, Meta Biomed, Korea) through a modified single-cone approach supplemented by auxiliary cones. Access cavities were temporarily closed with a temporary filling material (MD- TEMP, Meta Biomed, Korea). Postoperative instructions were provided, for pain control, participants received 400 mg of Ibuprofen as a rescue analgesic. Final coronal restorations were performed by specialists within the operative or prosthodontics departments at the Egyptian Russian University.

### Post-treatment pain evaluation

Each participant received a sheet containing the numerical rating scale (NRS-11), with “0” representing no pain and “10” representing the worst possible pain. Participants were instructed to use a chart to record pain intensity and frequency of analgesic intake at 6 h, 12 h, 24 h, the second day, and the third day after root canal treatment. All 60 patients completed their pain diaries in full, and data completeness was confirmed at the recall visit. No missing entries were identified.

### Outcomes

The primary outcome measured was to compare the impact of medical grade versus commercial sodium hypochlorite on post-treatment pain levels. Pain levels were recorded using an 11-point NRS. The percentage of pain reduction was calculated for each follow-up interval using the formula: [(Preoperative NRS - Postoperative NRS) / Preoperative NRS] *X* 100. This normalized measurement allowed for the assessment of relief relative to each patient’s unique baseline pain.

### Chemical verification

To ensure the standardization of the irrigation protocol, Chemical verification, via iodometric titration and pH analysis was conducted, after the study completion, at the Faculty of Pharmacy, Egyptian Russian University, under the supervision of a specialist in analytical chemistry (E.D.). Titration analysis was conducted on retained batch samples approximately six weeks after the conclusion of the clinical phase. During the interim, all solutions were maintained in their original, sealed, opaque containers at ≃ 25 °C, mirroring the storage conditions used throughout the trial. Two fresh, unopened bottles from the same batches used during the trial were analyzed: a medical-grade solution (JK Dental Vision, Egypt; Lot No. 037, MFG 08/2025) and a domestic-grade solution (Clorox, Egypt; Batch No. B-40, MFG 17/07/2025).

The concentration of free available chlorine (FAC) in the sodium hypochlorite solutions was verified using iodometric titration. A 10 ml sample of the solution was added to an Erlenmeyer flask containing 10 ml of distilled water, 2 g of potassium iodide (KI), and 5 ml of 10% acetic acid to liberate iodine. The resulting solution was then titrated against a standardized 0.1 N sodium thiosulfate (Na2S2O3) solution using a 1% starch indicator until the blue-black color disappeared (the endpoint). The FAC percentage was calculated according to the following equation:

$$\:\boldsymbol{F}\boldsymbol{A}\boldsymbol{C}\:\boldsymbol{\%}\:=\frac{\left[\boldsymbol{E}.\boldsymbol{P}\:\times\:\:\boldsymbol{S}\boldsymbol{t}\boldsymbol{a}\boldsymbol{n}\boldsymbol{d}\boldsymbol{a}\boldsymbol{r}\boldsymbol{d}\boldsymbol{i}\boldsymbol{z}\boldsymbol{a}\boldsymbol{t}\boldsymbol{i}\boldsymbol{o}\boldsymbol{n}\:\boldsymbol{F}\boldsymbol{a}\boldsymbol{c}\boldsymbol{t}\boldsymbol{o}\boldsymbol{r}\:\times\:\:\boldsymbol{E}\boldsymbol{q}\boldsymbol{u}\boldsymbol{i}\boldsymbol{v}\boldsymbol{a}\boldsymbol{l}\boldsymbol{e}\boldsymbol{n}\boldsymbol{t}\:\boldsymbol{F}\boldsymbol{a}\boldsymbol{c}\boldsymbol{t}\boldsymbol{o}\boldsymbol{r}\:\times\:100\right]}{\boldsymbol{V}\:\boldsymbol{S}\boldsymbol{a}\boldsymbol{m}\boldsymbol{p}\boldsymbol{l}\boldsymbol{e}}$$

Where:

- *E.P* is the volume of sodium thiosulfate consumed at the endpoint.
- *Standardization Factor* (0.98) accounts for the actual normality of the titrant.
- *Equivalent Factor* (0.03545) represents the grams of chlorine equivalent to 1 ml of 1 N sodium thiosulfate.
- *V sample* is the volume of the sodium hypochlorite sample (10 ml).

The alkalinity of the solutions was measured using a calibrated digital pH meter (JENWAY, 3505 Bibby scientific, UK). The measurements were performed at room temperature (≈ 25 °C) to ensure consistency with the clinical environment where the irrigants were stored and utilized.

### Statistical methods

Data were analyzed using IBM SPSS (Statistical Package for the Social Sciences) version (SPSS Inc., Chicago, IL). Normal distribution was explored using Komlmogorov-Smimov test and Shapiro-Wilk test. Numerical data were described as median and range as it were non-normally distributed. Comparison between the two groups done using Mann-Whitney U test where *p* ≤ 0.05 was considered statistically significant. Categorical data were described as percentages and comparisons were done using Chi-square test.

## Results

Ninety- seven patients were screened for eligibility, of whom 60 enrolled in the trial. There were 0 dropouts, thus all patients’ data were analyzed (Fig. 1). The study population comprised 30 females and 30 males, aged 29 to 47 years. A total of 34 first mandibular molars and 26 s mandibular molars were included. Baseline characteristics are represented in (Table 1), with no statistically significant differences observed between groups (*p* ≥ 0.05). A statistically significant difference in postoperative pain scores between the two groups was observed only at 6 h (*p* < 0.005) and 12 h (*p* = 0.023) postoperatively. Pain levels progressively decreased over time, reaching zero by the third day after treatment (Table 2). While the two groups had a non-significant difference in the intensity of preoperative pain (*p* = 0.684), there was a statistically significant difference in the percentage of pain reduction at 6 (*p* < 0.005) and 12 (*p* = 0.023) hours post-operatively (Table 3).

All 60 participants completed their pain diaries, which included records of rescue analgesic (400 mg Ibuprofen) consumption. Statistical analysis revealed no significant difference in the frequency or amount of analgesic intake between Group A and Group B (*p* > 0.05). By the third day, pain scores in both groups reached zero, and analgesic consumption ceased.

The results of iodometric titration and pH analysis revealed that the medical-grade solution exhibited a Free Available Chlorine (FAC) of 4.6% (4.83% NaOCl) with a pH of 12.29 while the domestic-grade solution demonstrated an FAC of 5.84% (6.13% NaOCl) and a pH of 12.1.

**Table 1:** Summary of participant baseline characteristics

	Group A	Group B	P-value
Age			
Median (range)	37.5 (29-47)	38.5 (30-44)	0.912 ns
Gender			0.371 ns
Male n (%)	12 (40%)	18 (60%)	
Female n (5)	18 (60%)	12 (40%)	
Tooth type			
First molar n (%)	19 (63.3%)	15 (50%)	
Second molar n (%)	11 (36.7%)	15 (50%)	0.435 ns

*Ns* Non-significant

**Table 2:** Statistical summary of numerical rating scale (NRS) results, displaying median values, ranges (minimum–maximum), and intergroup significance (*p*-values) for both study groups

	Group A (Domestic-grade NaOCl)	Group B (Medical-grade NaOCl)	*P*-value 1
Preoperative	8 (5–10)	7.5 (6–10)	0.684 ns
6 h postoperative	3 (1–8)	0	< 0.005 *
12 h postoperative	1.5 (0–6)	0	0.023 *
24 h postoperative	0 (0–2)	0 (0–3)	1 ns
48 h postoperative	0 (0–1)	0	0.481 ns
72 h postoperative	0	0	1 ns
*P*-Value 2	< 0.05 *	< 0.05 *	

*Ns* Non-significant

*P*-value 1 inter-group comparison, *P*-value 2 intragroup comparison

*: significant with *P*-value ≤0.05

**Table 3:** Percentage of pain reduction among groups:

	Group A (Domestic-grade NaOCl)	Group B (Medical-grade NaOCl)	*P*-value
6 h postoperative	57.3183 (20-85.71)	100	< 0.05 *
12 h postoperative	80.3571 (40–100)	100	0.023 *
24 h postoperative	100 (75–100)	100 (57.14–100)	0.971 ns
48 h postoperative	100 (87.5–100)	100	0.481 ns
72 h postoperative	100	100	1 ns

*Ns* Non-significant

The percentage of pain reduction was calculated for each follow-up interval using the formula: [(Preoperative NRS - Postoperative NRS) / Preoperative NRS]*X*100

*: significant with *P*-value ≤0.05

## Discussion

Pain management during and after endodontic treatment is a key clinical goal, as pain significantly influences patient comfort, cooperation, and the overall treatment experience. Effective pain control is therefore essential for successful endodontic outcomes [10]. Several intraoperative factors contribute to post-operative pain, and an important one is the type of irrigant used during treatment. Irrigants may irritate the periapical tissues, particularly if they are extruded beyond the apical foramen or used at high concentrations, causing periapical inflammation. Moreover, the chemical properties, cytotoxicity, and interaction of those irrigants with vital tissues can influence the intensity of post-operative pain, making careful selection and handling of irrigants essential to a pain-free treatment experience [11–13].

The effects of NaOCl, the most popular endodontic irrigant, on postoperative pain were studied at different temperatures [14, 15] and concentrations [13, 16] and in single- and multiple-visit settings [4, 13]. However, the effect of the particular formulation used, whether domestic-grade or medical-grade, on postoperative pain was never tested in the literature. Despite the uncertainty around the stability and concentration of domestic-grade NaOCl formulations, which could affect their cleaning ability and safety for use [6, 7]. Therefore, this clinical trial was designed to investigate the influence of commercial household bleach (domestic-grade NaOCl) and medical-grade NaOCl on the severity of postoperative pain in individuals diagnosed with symptomatic irreversible pulpitis of the mandibular molars.

The current study is a randomized, double-blind controlled clinical trial incorporating patient-reported outcome measures to provide the best possible approach for reducing selection bias. Blinding of participants and operators was employed to minimize both performance and detection bias, while the risk of attrition bias was eliminated due to absence of dropouts. Moreover, the successful randomization in the current study, as reflected by the lack of any significant difference between groups in all baseline data, including the intensity of preoperative pain, together with the set of specific inclusion criteria has minimized the effect of confounding factors [17].

Mandibular molars were selected in this study since they were reported to have the highest postoperative pain levels [18], and also to reduce the confounders by limiting the treatment to only one tooth type. All patients receiving any kind of NSAIDs 12 h before treatment were excluded to minimize the influence of those meds on diagnosis, intraoperative or postoperative pain [19]. NaOCl was used at half concentration (≈ 2.5%) since current evidence supports lower concentrations to achieve optimal disinfection and minimize detrimental effects on root dentine, as well as the risk of extrusion and postoperative pain commonly associated with higher concentrations [4, 16]. Since postoperative pain can be a direct consequence of debris and chemicals extrusion, irrigation was done using a freely movable, 29-gauge, side vented needle with maximum penetration depth set at 1 mm from the working length at a flow rate of 3 mL per minute in order to avoid any risk of unwanted extrusion that could confound the results [20, 21].

In the current study, patients reported their postoperative pain intensity on an NRS, which had 11 points, ranging from 0 (no pain at all) to 10 (the worst pain possible). All patients received a thorough explanation and training on how to record their pain levels at the specified time points, and they also recorded their preoperative pain level in front of the operator. The NRS was selected as the measurement tool since it was reported to be less complicated than Visual analogue Scales (VAS) yet more accurate than Verbal rating scale (VRS) [22, 23]. The time points were selected since postoperative pain was reported to be most prevalent at the first two days after treatment, hence the 6, 12, 24, and 48 h postoperative follow up time points [24, 25]. A 72-hour postoperative time point was added to further follow the course of postoperative pain.

The current investigation revealed that patients in the domestic-grade NaOCl group suffered significantly higher postoperative pain at 6 and 12 h postoperatively. These findings may be attributed to two primary underlying factors: concentration variability and chemical composition. Regarding concentration, domestic-grade preparations often lack a precise concentration label; the specific brand used in this study stated < 5 on the container, meaning the precise amount of free available chlorine (FAC), the active ingredient necessary for disinfection and tissue dissolution [26], was initially unverified for this group.

Subsequent chemical verification via iodometric titration, performed on fresh samples of the same batches used during the trial, confirmed this variability. The domestic-grade NaOCl (Batch B-40) exhibited an FAC of 5.84% (equivalent to 6.13% NaOCl), whereas the medical-grade NaOCl (Lot 037) exhibited an FAC of 4.6% (equivalent to 4.83% NaOCl). This confirms that the FAC concentration in the domestic-grade group significantly exceeded that of the medical-grade group. The lower stability and inconsistent levels of FAC in domestic-grade NaOCl compared to medical-grade NaOCl specialized for endodontic use have been documented in previous in-vitro studies [6, 26, 27]. Although higher NaOCl concentrations are associated with better antimicrobial efficacy, their use entails an increased risk of adverse effects, including irrigant extrusion, greater cytotoxicity, and higher postoperative pain [13, 28, 29].

Regarding chemical purity, medical-grade NaOCl is specifically formulated for dental use, involving precise filtration and buffering to maintain chemical stability. Conversely, domestic bleach is an industrial product containing surfactants and fragrances designed to lower surface tension [30]. This reduction in surface tension potentially increases the volume of irrigant contacting periapical tissues, facilitating deeper penetration into the periradicular bone and increasing cytotoxicity independent of the hypochlorite concentration.

Our post-hoc analysis confirmed a higher alkalinity in the medical-grade solution (pH 12.29) compared to the domestic grade (pH 12.1). In the context of endodontic irrigants, these results suggest a functional plateau in caustic efficacy. Because both solutions exceeded the critical pH threshold of 11.5, the point at which maximum protein denaturation and tissue dissolution typically occur, the slight variance in alkalinity was clinically negligible. This aligns with the established principle that while a high pH is required for tissue dissolution, there exists a functional plateau in caustic efficacy beyond the threshold of pH 11.5–12.0 [31].

At this extreme alkalinity, the slight variance between the domestic (12.1) and medical (12.29) solutions is neutralized by the buffering capacity of dentin, leaving the higher postoperative pain response in the domestic-grade NaOCl group to be explained by the significantly higher free available chlorine (FAC 6.13% vs. 4.83%) [32]. The superior oxidative potential of the higher chlorine concentration, likely compounded by industrial surfactants that promote the ‘spread’ of the irrigant beyond the apical foramen, appears to be the primary etiologic factor for the observed periapical inflammatory response [28, 29].

It is also worth noting the clinical significance of the statistical findings. At 12 h, the median pain score in the domestic bleach group was 1.5 (mild pain), compared to 0 (no pain) in the medical-grade group. While statistically significant, a score of 1.5 is considered tolerable. However, the complete absence of pain in the medical-grade group suggests that using a professional formulation may prevent even the “mild discomfort” that is often accepted by endodontists as a normal postoperative occurrence [24, 33].

This study has several limitations that should be acknowledged. First, the trial was retrospectively registered, which represents a deviation from the ideal prospective registration recommended by CONSORT and ICMJE guidelines, however, ethical approval was strictly obtained prior to patient enrollment. Second, while the sample size calculation was based on a pilot-derived effect size of 1.23, it is recognized that pilot data can sometimes lead to an overestimation of true clinical effects. To mitigate this, the study employed a high power of 98%, yielding 30 participants per group, a figure that substantially exceeds the 17 participants per group required at a standard 90% power. This surplus in sample size ensures the statistical reliability of the findings. Regarding chemical analysis, titration was performed post-hoc on identical manufacturer batches rather than on the specific clinical aliquots. While this verified the stability of the lot used, future research should ideally utilize real-time, point-of-care titration to account for minor storage-related fluctuations. Even though limiting the study to mandibular molars and a single experienced operator ensured high internal validity, this focus, along with the specific irrigant formulations used, may limit generalizability across diverse tooth morphologies, product profiles, and practitioner experience levels. Furthermore, as the study was restricted to symptomatic irreversible pulpitis, these findings should be applied with caution to cases of pulpal necrosis or retreatment, which present distinct inflammatory and microbial environments.

## Conclusions

Within the limitations of this clinical trial, the use of domestic-grade sodium hypochlorite (NaOCl) for single-visit root canal treatment was associated with significantly higher early postoperative pain compared to the medical-grade formulation. Chemical verification revealed that the domestic-grade formulation had a higher concentration of NaOCl and free available chlorine (FAC), which likely contributed to increased periapical irritation. While pain levels were generally mild, the complete absence of early postoperative pain in the medical-grade group suggests a distinct clinical advantage in patient comfort. Consequently, standardized medical-grade NaOCl is highly recommended to ensure controlled concentrations and a more predictable postoperative course. 

## Supplementary Information

Supplementary Material 1.

## Data Availability

The datasets used and/or analyzed during the current study are available from the corresponding author on reasonable request.

## Abbreviations

NaOCl: Sodium hypochlorite
NRS: Numerical rating scale
CONSORT: Consolidated Standards Of Reporting Trials
FAC: Free available chlorine
PDL: Periodontal ligament
NiTi: Nickel-titanium
EDTA: Ethylenediaminetetraacetic acid
NSAID: Non-steroidal anti-inflammatory drug
SPSS: Statistical Package for the Social Sciences
VAS: Visual analogue scale
VRS: Verbal rating scale
pH: Potential of hydrogen
